# Estimating the cost of care giving on caregivers for people living with HIV and AIDS in Botswana: a cross-sectional study

**DOI:** 10.1186/1758-2652-13-14

**Published:** 2010-04-20

**Authors:** Njoku O Ama, Esther S Seloilwe

**Affiliations:** 1 Department of Statistics, University of Botswana, 4775 Notwane Road, Gaborone, Botswana; 2 Department of Nursing Education, University of Botswana, 4775 Notwane Road, Gaborone, Botswana

## Abstract

**Background:**

Community home-based care is the Botswana Government's preferred means of providing care for people living with HIV (PLHIV). However, primary (family members) or volunteer (community members) caregivers experience poverty, are socially isolated, endure stigma and psychological distress, and lack basic care-giving education. Community home-based care also imposes considerable costs on patients, their caregivers and families in terms of time, effort and commitment. An analysis of the costs incurred by caregivers in providing care to PLHIV will assist health and social care decision makers in planning the most appropriate ways to meet future service needs of PLHIV and their caregivers.

**Methods:**

This study estimated the cost incurred in providing care for PLHIV through a stratified sample of 169 primary and volunteer caregivers drawn from eight community home-based care groups in four health districts in Botswana.

**Results:**

The results show that the mean of the total monthly cost (explicit and indirect costs) incurred by the caregivers was $(90.45 ± 9.08) while the mean explicit cost of care giving was $(65.22 ± 7.82). This mean of the total monthly cost is about one and a half times the caregivers' mean monthly income of $66.00 (± 5.98) and more than six times the Government of Botswana's financial support to the caregivers. In addition, the cost incurred per visit by the caregivers was $15.26, while the total expenditure incurred per client or family in a month was $184.17.

**Conclusions:**

The study, therefore, concludes that as the cost of providing care services to PLHIV is very high, the Government of Botswana should substantially increase the allowances paid to caregivers and the support it provides for the families of the clients. The overall costs for such a programme would be quite low compared with the huge sum of money budgeted each year for health care and for HIV and AIDS.

## Background

There are various models of home-based care for people living with HIV (PLHIV). Each has a different delivery scheme, offers a mix of services, has different staff and outreach, and emphasizes different programmes [1]. The models include facility-based, community-based and integrated approaches [1,2]. The facility-based programmes often focus on the medical aspects of care and involve teams that include health professionals who can provide higher levels of care. Community home-based care (CHBC) programmes provide psycho-social support for PLHIV and their families, and deliver services primarily through volunteer networks in the community [1]. The integrated model emphasizes collaboration between a number of partners, and the programmes are often integrated into some health care services [3].

Community home-based care, which informs our present work, is the preferred health care delivery model used by the Government of Botswana to provide care for PLHIV. CHBC has been defined as the care given to an individual in his or her own environment (home) by his or her family. The family members are supported by skilled welfare officers and communities to meet not only the physical and health needs, but also the spiritual, material and psycho-social needs of the clients [4,5].

As the number of people living with HIV and AIDS increases, the gap between the demand for and the availability of health care services continues to widen. Relying mainly on the family and community as caregivers, CHBC has become a significant contributor in the treatment, care and support of those infected and affected by HIV and AIDS.

CHBC primary (family members) or volunteer (community members) caregivers for PLHIV [6] experience poverty, are socially isolated, endure stigma and psychological distress, and lack basic care-giving education [7]. CHBC also imposes considerable costs on patients, their caregivers and families in terms of time, effort and commitment. The costs have been classified by Gold *et al *[8] as "direct medical costs" (such as those for medication, physician fees, hospitalizations and office visits) and "direct non-medical costs" (such as transportation, dietary supplements, labour costs and lost wages associated with time spent on care giving).

The direct non-medical costs arise from replacement of employees who quit their employment due to their care-giving responsibilities, absenteeism costs, and costs due to partial absenteeism, workday interruptions and supervision of employed caregivers [9]. Direct non-medical costs will continue to grow as more and more people with HIV and AIDS are transferred from the hospital to the home setting, and as the burden of caring for the client falls increasingly on families and volunteer caregivers.

An estimation of the cost of care giving is very important in a comprehensive economic analysis. A number of studies [8,10,11] have shown that costs incurred by care recipients and unpaid caregivers account for a significant proportion of the total healthcare expenditure, while Moalosi *et al *[12] notes that the cost of care giving to caregivers of tuberculosis and lung disease patients has remained substantial in relation to the income levels of home-based caregivers.

Yet many economic evaluations do not include these costs [11]. It was reported by McDaid [13] that the variation in the proportions of the total costs of care giving represented by informal care, which includes home nursing and domiciliary care, as well as caregiver time in selected studies for people living with Alzheimer disease in the community, was between 36% and 85%.

This paper, which follows from a study by the authors [14] between June and September 2008, focuses on the costs incurred in care giving by primary and volunteer caregivers for PLHIV under the CHBC programme. It adopts the opportunity cost method [11,15-17] to value the time investment of caregivers in informal care and to determine the direct non-medical costs the caregivers incur in providing care to PLHIV. These costs were examined within the context of: increased cost of living; decreased income due to loss of jobs and/or job opportunities; transport to and from the place of giving care as well as to health facilities; death and/or funeral costs; financial donations to the clients or their families; disruption of their daily routines, social relationships and emotional well-being; and expectations about care giving.

Based on the caregivers' responses, this paper: describes the characteristics of the caregivers; estimates the direct non-medical costs of care giving; estimates the average cost incurred by caregivers per home visit and per client and/or family; and compares the cost incurred with the amount of government allowances given to the caregivers.

An analysis of the cost incurred by caregivers in providing care to PLHIV is critical. It will assist health and social care decision makers in planning the most appropriate ways to meet future service needs of PLHIV and the caregivers. Public health departments in Botswana will be able to develop interventions for the improvement of the CHBC programme.

## Methods

### Design

The study was cross sectional. It used quantitative methods in obtaining information from the caregivers. A three-stage stratified sampling method was used in the study. The health districts, community home-based groups and caregivers constituted the three strata. Four health districts (two urban and two rural) were randomly selected from the list of 16 urban and rural districts (four urban and 12 rural) that had established community home-based care groups.

From each of the sampled health districts, two community home-based care groups were randomly selected and the list of caregivers obtained from the community home-based care coordinators. A list of caregivers was kept in each community home-based care office, and this formed the sampling frame for the study. The sample size of caregivers was allocated to the eight community home-based care groups using proportional allocation to size. The caregivers to be interviewed were then selected randomly.

### Study questions

The study answered the following questions:

(i) How much, on average, does a volunteer caregiver spend on transportation when he or she visits the client or to take her or him to the hospital and/or clinic for treatment?

(ii) What is the estimated cost incurred by a caregiver in helping the family of the client and in providing food for himself or herself during each visit?

(iii) What is the estimated cost of providing other services (bathing client, washing clothes, planting vegetables, cleaning the home, etc.) to the client?

### Setting and sample

This study was conducted between June and September 2008 and was supported by funding from the Office of Research and Development, University of Botswana. It covered Gaborone, Kweneng East, Selibe Phikwe and Bobirwa health districts in Botswana. It targeted 498 caregivers from the eight sampled community home-based care groups. Using the sample size calculator programme [18] that allows for 95% confidence (and an error margin of 4%), and that posits that the response from the sampled population would be the same as that of the entire population, the estimated sample size for the study was 272. This number was allocated to the eight sampled community home-based care groups using probability proportional to size. Simple random sampling, using the list of caregivers kept at the community home-based care offices as the sampling frame, was employed in identifying the specific caregivers to be interviewed.

### Instruments for the study

The research instruments used in this study were the questionnaire developed by the authors, drawing experience from relevant literature [19], and a semi-structured interview guide. The questionnaire contained questions about the caregivers' demographic characteristics, their opinions on the services they rendered to the clients and the average time they spent in providing each service per week, the various costs they incurred, and the burdens they experienced while delivering care. Other areas investigated were the quality of care provided and the facilities they were provided with for caring for the clients. Answers to some of the questions were provided on a five-point scale; in other cases, the questions were open ended and gave the caregivers an opportunity to express their own opinions on a number of issues.

### Psychometric properties of the questionnaire

The quality and content validity of the questionnaire was assessed by staff in the Nursing and Statistics departments at the University of Botswana, while the staff of the Community Home-Based Care Programme of the Ministry of Health, Botswana, assessed the protocols for the use of appropriate terminologies and the appropriateness of selected groups in the sample to be studied. The questionnaire was later tested on a sample of 20 caregivers selected from a CHBC group in Gaborone, different from those to be studied, for content validity and quality and internal consistency. The Cronbach alpha was calculated as 0.89.

### Data collection

The questionnaire was administered to the sampled caregivers by trained research assistants at their homes or at the community home-based care offices. Data were collected from 169 caregivers (about 62% of the targeted sample) because some of them were reluctant to participate in the study and because some of them were not available after several visits by the research assistants within the period of the survey.

This response percentage is reasonably high and very much higher than those obtained by Sevick *et al *[20] (19.7%), and according to Keeter *et al *[21], creates insignificant problems in the analysis of the results (see also Visser *et al *[22]) The interview guide was used in collecting information from the coordinators of the CHBC groups on caregivers' perceived experiences.

### Ethical consideration

The Health Research Committee of the Ministry of Health, Botswana, considered and approved the protocols for the study as meeting all ethical considerations. The purpose of the study was explained to the caregivers by trained research assistants before questionnaire administration. The caregivers were informed that participation in the study was voluntary, that there was no payment for participation, and that they were free to withdraw from participating at any time. They were assured of the confidentiality of the information obtained as the questionnaire was coded to ensure anonymity. Each caregiver willing to participate in the study gave a written consent.

### Estimation of cost incurred by caregivers

The costs that have been included in this study are those borne by the primary and volunteer caregivers. They are: the value of time that a member of the household or volunteer caregiver spent taking care of the PLHIV; cost incurred in transport to and from the place of care giving and cost of the caregiver feeding himself or herself during each visit, particularly when care giving extends over a long period or overnight; and the cost incurred in taking the client to the hospital or clinic or in assisting the family of a client during a funeral.

These costs have been described by Gold *et al *[8] as direct non-medical costs. However, because drugs (including antiretrovirals) are usually given free of charge in Botswana, costs incurred by caregivers in assisting the client or family of the client in purchasing drugs have been excluded from the cost items [23].

The direct non-medical costs incurred by caregivers have been split into explicit and indirect costs (B. Ralph, pers. comm.). The explicit costs include: cost of transportation to and from the place of delivering care; transporting the client to a hospital or clinic for check up and treatment; assistance to families of clients to establish income-generating ventures; money spent in providing food to the client and family members; such gifts as clothes and support during funerals; and money that the caregiver spent in providing food for himself or herself during visits to the client. Data needed to estimate these costs were collected in answers to questions that solicited from the caregivers how much they spent on transport, how much they spent on supporting the client and family, and how much they spent on providing food for themselves during visits.

The opportunity cost of giving care to PLHIV was estimated based on the time spent in providing various services to the client and family. These services include encouragement of the clients, keeping the clients company, collecting water, washing clothes, preparing meals, collecting drugs from the clinics or hospitals, counselling the clients, cultivation of crops and growing vegetables for clients and families, and arranging access to food baskets. Opportunity cost refers to the market-based earning capacity of the caregiver, which is determined by her or his education and skill [20]. It has also been referred to as the implicit costs (B. Ralph, pers. comm.). It has often been recommended that the opportunity cost method or replacement cost method be used to value the time investment in informal care because of its relatively straightforward application [24-27].

Conceptually, the opportunity cost method values informal care according to the following equations:(1)

β_i _is the time spent on care tasks by caregiver *i*, and w_i _is the net market wage rate of i^th ^caregiver [27]. If the i^th ^caregiver is unemployed, a proxy for w_i _is used, e.g., a modified opportunity cost method to find out the reservation wage of the caregiver.

Estimating the opportunity cost of giving care by the caregiver takes into consideration the caregiver's educational status and the income the caregiver would have earned if he or she had not been providing care to PLHIV. For those employed, their current salaries were used in multiplying the time spent to provide care. For the unemployed, the statutory minimum wage for the private and parastatal sectors ($0.40/hour) [12] was used as the hourly income of the caregiver. This proxy wage was used to multiply the time spent in care giving to provide the estimated income loss per month due to providing care [28].

The reliability of the cost estimates was 0.80 and the total cost of providing care by the caregiver is given by:(2)

### Data analysis

Data were captured using the Statistical Package for the Social Sciences (SPSS) computer programme. All variables, including the responses to the open-ended questions, were coded before being captured using the programme. Data were analyzed using descriptive measures, such as percentages, means, standard deviation and inferential statistics, such as the t-test. The t-test was used to determine if there were significant differences in mean expenditure between employed and unemployed caregivers, and between rural and urban districts. Graphical representations helped to further illustrate the results obtained. The results of the study were disseminated through departmental seminars.

## Results

### Characteristics of caregivers

Of the 169 caregivers who participated in this study, 91% were female and 9% were male. A little over 50% were older than 40 years and about 39% older than 50. Forty-four percent of the caregivers were in the range of 21 to 40 years. The majority of the caregivers (73%) were volunteers, while 27% were primary caregivers. About nine in every 10 caregivers (93%) had secondary school certificates or less. Forty-three percent of them had been trained to provide care. While 77% of the caregivers were unemployed, 23% were employed. An overwhelming majority of the caregivers (91%) earned monthly income below one thousand pula (about US$140) and about 57% had provided care for, at most, four years.

### Services rendered to clients by caregivers

**Figure 1:**
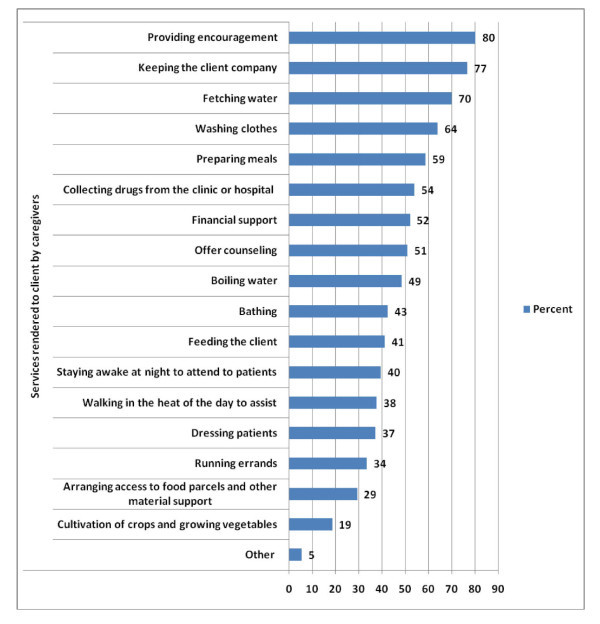
**Percentage of caregivers offering identified home based care services to clients**.

The types of services the caregivers provided to the clients are shown in Figure 1. The figure shows that 80% of the caregivers primarily provided encouragement to clients, 77% kept the clients company, 70% collected water, 64% washed clothes, 59% prepared meals, 54% collected drugs from the clinics or hospitals, 52% supported the clients financially, and 51% counseled the clients. Arranging access to food baskets (29%) and cultivation of crops and growing vegetables for clients and families (19%) were the least executed services.

### Costs incurred by caregivers

The results of the costs incurred by caregivers are summarized in Tables 1 and 2 by the employment status and location where the caregiver provides care.

**Table 1:** Cost ($) incurred by caregivers (monthly) in providing care to PLHIV

Employment status of caregiver		Amount spent ($) in a month to support client and family (A)	Amount spent in a month to take care of himself/herself during visits (B)	Amount spent ($) on transport in a month to give care (C)	Total explicit cost ($) (D) = A +B+C	Opportunity cost ($) of providing care by caregivers (E)	Total cost ($) of care giving on caregivers (D+E)
Employed	Mean	46.02	26.23	18.91	91.16	47.33	138.5
	N	39	39	39	39	39	39
	Std. error	6.86	14.08	13.39	14.6	17.28	32.38

Unemployed	Mean	42.31	7.49	7.63	57.43	18.6	76.03
	N	130	130	130	130	130	130
	Std. error	4.73	0.66	0.67	4.8	1.85	5.53

Total	Mean	43.16	11.82	10.24	65.22	25.23	90.45
	N	169	169	169	169	169	169
	Std. error	3.96	3.44	3.52	7.82	4.3	9.08

**Table 2:** Cost ($) incurred in providing care by caregivers classified by urban and rural districts

Location where caregiver provides care		Amount spent ($) in a month to support client and family (A)	Amount spent in a month to take care of himself/herself during visits (B)	Amount spent ($) on transport in a month to give care (C)	Total explicit cost($) D = A+B+C	Opportunity cost ($) E	Total cost ($) of care giver on caregivers (D+E)
Urban	Mean	50.04	19.01	13.99	83.05	23.93	106.98
	N	68	68	68	68	68	68
	Std. error	6.86	14.08	13.39	16.68	4.69	17.44

Rural	Mean	38.53	6.97	7.71	53.21	26.11	79.32
	N	101	101	101	101	101	101
	Std. error	5.36	1.53	2.73	6.54	6.48	9.55

Total	Mean	43.16	11.82	10.24	65.22	25.23	90.45
	N	169	169	169	169	169	169
	Std. error	3.96	3.44	3.52	7.82	4.3	9.08

US$1 = P7.68 (June/July 2008)

Table 1 shows that the mean monthly expenditure incurred by the employed caregivers was $138.50 ± 32.38 (that is, between $106.12 and $170.88), while the mean expenditure incurred by unemployed caregivers was $76.03 ± 5.53 (between $70.50 and $81.56). Generally, the employed caregivers spent more on all items than the unemployed. However, the difference in their expenditure regarding financial support to the client and family is not significant (t = 0.32; p > 0.05). Overall, the mean cost incurred by the caregivers per month in providing care was $90.45 ± 9.08 (that is between $80.37 and $99.53), while the overall explicit cost of care giving by the caregivers was $65.22 ± 7.82 (between $57.40 and $73.04).

The study further revealed that the total number of visits made by 169 caregivers in a month to the 83 clients studied was 1002 or an average of six visits per month per caregiver. The cost incurred per visit by the caregivers was $15.26, while the total expenditure incurred by caregivers per client and/or family in a month was $184.17.

Table 2 shows that the there are no significant differences in the explicit costs of providing care by the caregivers in the urban and rural districts (t = 1.29, p-value > 0.05) and also in the opportunity costs (t = - 0.195; p-value > 0.05).

### Caregivers' support

When the caregivers were asked who covered their expenditure during each visit, the responses showed that 70% supported themselves, while about 9% relied on the allowance of $15.26 given to them by the government and 5% used donations from people, including their relatives. In addition, while 100% of the caregivers in Bobirwa district were self-supported, the corresponding percentages from Selibe Phikwe, Gaborone, and Kweneng East districts were 85, 55 and 19, respectively. About one out of every four caregivers from Gaborone district received an allowance of $15.26 every month from the Government of Botswana, while 5% in Selibe Phikwe health district received allowances, and none in Bobirwa and Kweneng East received allowances.

## Discussion

One of the most frequently mentioned challenge in care giving is that families do not have sufficient resources to cover the cost of caring for sick people [29]. Caregivers are therefore compelled to provide their clients with supplies, such as food and washing soap, support them by transporting them to hospitals or clinics to collect their drugs, and cover the cost of caring for the clients [29]. This study showed that caregivers' monthly mean earning was $66.00 ± 5.98, yet the mean explicit cost incurred in providing care was $65.22 ± 7.82. This implies that the caregivers spent almost their entire income on care giving and must have sustained themselves in their care-giving activities with support from government (9%) and donations from other people, including relatives and community members (5%).

This finding illustrates the fact that community home-based care is not a cheap endeavour and that the cost of care giving has only shifted from the government to the families and caregivers, who now incur enormous expenditure in care-giving activities. This calls for an urgent public health intervention to provide financial and material support for the caregivers so that they are not demoralized in rendering care services to their clients.

This view is also in line with that of Phaladze [30] and others [31] who showed that caregivers need financial and material supplies to maintain their morale. According to Seloilwe [32], some respondents suggested that caregivers should be paid to provide services. One female beneficiary noted that she would like volunteers to be hired full time so that they could be paid for the good job that they are doing.

The study found that the cost of providing care per client per month was $184.17, which is significantly higher than the cost per client in the CHBC programme in Rwanda, where monthly costs per client range from $12.75 to $24.53 [1]. This high cost incurred per client per month and the high cost per visit ($15.26) may account for the reduced number of visits per client. In Zimbabwe, for instance, when the cost per home-care visit decreased from $10 to $1 as the programme expanded, the number of clients and visits also increased and the programme became more efficient [33]. Both Hansen *et al *[33] and Foster *et al *[35] have shown that reduced cost per visit leads to an increased number of visits.

Currently, the Government of Botswana gives a monthly allowance of $15.26 to the caregivers. When compared with the mean cost of providing care, as evidenced in this study, it is clear that there is an urgent need for adequate financial incentive to motivate those already providing care to PLHIV in Botswana and to persuade people to take up care-giving activities. Providing incentives, such as mealie meal and food baskets and loans for income-generating activities, and lending a sympathetic ear to their plight will help boost the morale of caregivers and attract others to care giving [14].

The finding that the government's financial support, small as it is, is either not evenly distributed to caregivers (those in districts such as Gaborone receive more) or not provided at all (such as in Bobirwa) points to the weakness of the public health intervention measures in Botswana. Such measures should ensure that equity is maintained in the distribution of support to caregivers, irrespective of where they provide care. Government should therefore harmonize support given to caregivers.

While CHBC may be seen as a cheap alternative mode of health care delivery, it is certainly not affordable for families and caregivers who provide it. Results indicate that families are actually struggling to make ends meet. Therefore, it is important for government to consider how best costs of providing care can be shared between them and those who provide care.

There are several limitations to this study. The study covered only eight community home-based groups selected from four health districts in Botswana. Although appropriate methods were used to collect the data, the study is, however, limited in its scope. The caregivers did not keep any notebook records showing the time spent daily or weekly on care giving. The study relied on information provided by caregivers. All the analyses in the study have been made on the assumption that the information provided was accurate and reflected the true perceptions of the caregivers. The authors do acknowledge that results of the studies are based on respondents' supplied information from eight CHBC groups selected from four health districts. Interpretations of the results are, therefore, limited to the studied areas, but the results do provide strong reasons for extending the study to other CHBC groups in the country.

## Conclusions

As the cost of providing care services to PLHIV is very high, the Government of Botswana should substantially increase the allowances paid to caregivers and the support it provides for the families of the clients. The overall costs for such a programme would be quite low compared with the huge sum of money budgeted each year for health care and for HIV and AIDS [35].

In addition, the government's financial support to caregivers should be evenly distributed to the caregivers in all the districts (rural or urban). This would lessen the possibility of high costs of visits to clients, of care giving being infrequent, and of this adversely affecting the objective of setting up the community home-based care programme.

## Competing interests

The authors declare that they have no competing interests.

## Authors' contributions

NOA designed the study, collected the data and analysed it, developed the manuscript including subsequent revisions of the manuscript. ESS participated in the initial development of the study, developed the instrument for the study, collected the data and made inputs into the various versions of the revised manuscript. Both authors read and approved the final manuscript.
